# The target/perpetrator brief-implicit association test (B-IAT): an implicit instrument for efficiently measuring discrimination based on race/ethnicity, sex, gender identity, sexual orientation, weight, and age

**DOI:** 10.1186/s12889-021-10171-7

**Published:** 2021-01-19

**Authors:** Maddalena Marini, Pamela D. Waterman, Emry Breedlove, Jarvis T. Chen, Christian Testa, Sari L. Reisner, Dana J. Pardee, Kenneth H. Mayer, Nancy Krieger

**Affiliations:** 1 Istituto Italiano di Tecnologia, Ferrara, FE Italy; 2 Harvard T.H. Chan School of Public Health, Boston, MA USA; 3 The Fenway Institute, Boston, MA USA

**Keywords:** Discrimination, Implicit measures, Implicit association test (IAT), Brief implicit association test (B-IAT), Race/ethnicity, Sex, Gender identity, Age, Sexual orientation, Weight

## Abstract

**Background:**

To date, research assessing discrimination has employed primarily explicit measures (i.e., self-reports), which can be subject to intentional and social desirability processes. Only a few studies, focusing on sex and race/ethnicity discrimination, have relied on implicit measures (i.e., Implicit Association Test, IAT), which permit assessing mental representations that are outside of conscious control. This study aims to advance measurement of discrimination by extending the application of implicit measures to multiple types of discrimination and optimizing the time required for the administration of these instruments.

**Methods:**

Between September 27th 2019 and February 9th 2020, we conducted six experiments (984 participants) to assess implicit and explicit discrimination based on race/ethnicity, sex, gender identity, sexual orientation, weight, and age. Implicit discrimination was measured by using the Brief-Implicit Association Test (B-IAT), a new validated version of the IAT developed to shorten the time needed (from ≈15 to ≈2 min) to assess implicit mental representations, while explicit discrimination was assessed using self-reported items.

**Results:**

Among participants (mean age = 37.8), 68.6% were White Non-Hispanic; 69% were females; 76.1% were heterosexual; 90.7% were gender conforming; 52.8% were medium weight; and 41.5% had an advanced level of education. Overall, we found implicit and explicit recognition of discrimination towards all the target groups (stronger for members of the target than dominant groups). Some exceptions emerged in experiments investigating race/ethnicity and weight discrimination. In the racism experiment, only people of Color showed an implicit recognition of discrimination towards the target group, while White people were neutral. In the fatphobia experiment, participants who were not heavy showed a slight implicit recognition of discrimination towards the dominant group, while heavy participants were neutral.

**Conclusions:**

This study provides evidence that the B-IAT is a valuable tool for quickly assessing multiple types of implicit discrimination. It shows also that implicit and explicit measures can display diverging results, thus indicating that research would benefit from the use of both these instruments. These results have important implications for the assessment of discrimination in health research as well as in social and psychological science.

**Supplementary Information:**

The online version contains supplementary material available at 10.1186/s12889-021-10171-7.

## Background

Exposure to discrimination produces severe consequences on health (for a recent review, see [1]). It can increase risk of sleep disorders [2–6], adverse physical and psychosocial conditions (e.g., cardiovascular disorders, psychological distress, and major depression [7–11]), as well as harmful coping behaviors (e.g., substance use, such as tobacco and alcohol [12–16]).

Given the relevant impact of discrimination on health, a crucial aspect for empirical research is to define best measures and methods to quantify exposure to discrimination [17–19] in order to accurately estimate the population at risk and prevent potential negative health outcomes.

Over the last two decades, studies assessing discrimination primarily relied on explicit measures (i.e., self-reports) [11, 17, 20, 21], which reflect conscious and controllable evaluations. Self-report data are thus potentially subject to intentional and social desirability processes [22] that might prevent people from accurately reporting discrimination if they think this could be viewed negatively by others or unsafe for them. For example, individuals may be aware of having been a target of discrimination but they may be unwilling to disclose this information because they do not want to present themselves as weak and vulnerable or place themselves in potential danger.

To address the limitations of explicit measures, to our knowledge only four recent studies [23–26] have assessed exposure to discrimination using the Implicit Association Test (IAT) [27]. The IAT is a widely-used and validated implicit measure that infers automatic and spontaneous mental representations that exist in memory [28]. Unlike explicit measures, the IAT assesses mental contents indirectly by measuring how quickly and accurately a person can categorize and associate stimuli related to two conceptual categories and two evaluative attributes. The underlying presumption is that categories and attributes that are strongly associated at a mental representation level show shorter latencies and fewer errors when classified together than when they are not [27, 29]. The IAT thus is thought to be less influenced by conscious intentions and social desirability processes and to capture constructs that are outside of intentional and direct control [28].

For example, in an IAT assessing exposure to racial/ethnicity discrimination, participants are asked to categorize stimuli representing the two conceptual categories – White people and Black people – and the two evaluative attributes – Target and Perpetrator – in two sorting conditions. In one condition, participants categorize stimuli representing the categories White people and Perpetrator with one response key, while categorizing stimuli representing Black people and Target by using another response key. In the other condition, participants categorize the same stimuli but with a different key configuration: this time stimuli of White people and Target are categorized with one key, whereas stimuli of Black people and Perpetrator are categorized with the other. Faster categorization in the first condition compared to the second condition indicates an implicit recognition of discrimination towards Black people than White people (implicit discrimination).

Studies using the IAT to measure exposure to discrimination have provided relevant insights into health research as well as in social and psychological sciences. For example, Carney et al. [23] employed the IAT to investigate the well-known person-group discrimination discrepancy (PGDD [30, 31];) typically observed on explicit measures. The PGDD refers to the tendency, showed by members of target groups, to report that other members of their social groups are discriminated against (group discrimination), but that they are not (person discrimination). Authors showed that although members of target groups do not explicitly report person discrimination, they do reveal person discrimination on the IAT. These results were observed in relation to sex and race/ethnicity discrimination [23]. In addition, a series of three studies used the IAT to investigate the relation between exposure to discrimination and health outcomes. They showed that implicit measures of exposure to racial discrimination were associated with smoking [25], elevated blood pressure, and risk of hypertension [24, 26]. In other words, stronger implicit discrimination predicted an increased risk of physical conditions and behaviors harmful for health.

Taken together, these findings indicate that implicit and explicit measures of exposure to discrimination are not equivalent and research thus would benefit by using implicit measures to have an assessment of exposure to discrimination that is less influenced by intentional and conscious processes, such as social desirability. Indeed, research exploring the effects of social desirability on explicit and implicit measures of discrimination, showed that explicit measures (but not implicit measures) are associated with social desirability [25]. Thus, investigating discrimination only by means of explicit measures might be inadequate because people may not accurately report exposure to discrimination at a conscious level.

The main goal of the present study is to advance measurement of exposure to discrimination by evaluating the application of implicit measures to different types of discrimination and by using a new brief validated version of these instruments to optimize the time required for their administration. Existing studies using the IAT for discrimination focused only on sex and race/ethnicity discrimination and employed exclusively the standard version of this instrument [23–26], which requires around 13 min for administration. To our knowledge, no studies have used implicit measures of exposure to discrimination in relation to other social groups or used the new brief version of the IAT (Brief-Implicit Association Test, B-IAT, [32, 33]) that only requires two minutes to be administered.

In this study, we performed such an investigation and conducted six experiments using the B-IAT to assess exposure to discrimination in relation to race/ethnicity, sex assigned at birth,^1^ gender identity, age, sexual orientation, and weight. In addition, to have a more comprehensive view of this phenomenon, we (1) measured exposure to discrimination also by means of explicit measures (explicit self-reports of exposure to discrimination) and (2) assessed implicit and explicit attitudes towards different social groups of interest in each experiment in order to evaluate the existence of a possible association between discrimination and social attitudes.

## Methods

### Participants

The present study included six experiments, one experiment for each of the six types of discrimination investigated (i.e., race/ethnicity, sex, gender identity, age, sexual orientation, and weight). The study protocol was approved by the institutional review board at the Harvard T.H. Chan School of Public Health (IRB 18–1128).

Between September 27th 2019 and February 9th 2020, a total of 984 participants completed at least one measure included in each experiment of the present study. Participants in all six experiments were United States (U.S.) citizens and residents, aged between 25 to 64 years. This age range was selected to focus on working-age adults, given the documented importance of work as a primary site for exposure to discrimination [1, 17, 20, 36], with this age group also having had the possibility of experiencing discrimination in other domains (e.g., at school, getting housing, from the legal system or police, getting medical care, getting a loan or mortgage, shopping, on the street or in a public setting, etc.) [1, 11, 17–21, 37].

All data were collected through the Project Implicit research website (https://implicit.harvard.edu). Participants were recruited from people who visited the Project Implicit website and volunteered for completing studies. The exception was for transgender participants in the transphobia experiment, who were actively recruited, given their lower representation among people visiting the Project Implicit website. Transgender participants were recruited by sending an invitation email to a number of local and national transgender-specific listservs and by posting an advertisement on social media (i.e., Facebook, Instagram, and Linkedln), selected via our work with Fenway Health Institute, a major U.S. community health center specializing in the healthcare of sexual and gender minority people.

Data collection stopped once each experiment reached 150 participants (i.e., 75 participants for each group of interest) with completed sessions in all the measures used. For example, in the sexism experiment, data collection stopped once we reached 75 female participants and 75 male participants with completed sessions. Post hoc power analysis showed that this sample allowed us to have a greater than 90% power at detecting an implicit or an explicit score significantly different from zero with an effect size of Cohen’s d = 0.25 and an alpha significance criterion of 0.05 (one-tailed t-test; G ∗ Power 3) [38].

### Procedure

In all experiments, each participant first filled out a request for key demographic items (listed below) and then completed four measures:

1. a Target/Perpetrator Brief Implicit Association Test (Target/Perpetrator B-IAT) to measure the strength of association between the two social categories of interest (e.g., in the racism experiment, *People of Color* vs. *White People*) and the attributes *target* and *perpetrator* (implicit discrimination),
2. a Good/Bad B-IAT to measure the strength of association between the two social categories of interest and the attributes *good* and *bad* (implicit attitude),
3. two self-reported items to measure the explicit self-reported group and individual discrimination (explicit discrimination),
4. a self-reported item to measure explicit attitudes towards the two social categories of interest (explicit attitude).

For each experiment, in the demographic questionnaire participants evaluated themselves as belonging either to the target or perpetrator group, and then completed the implicit and explicit instruments in relation to the type of discrimination at issue. For example, in the experiment for racial discrimination, participants who self-identified as being a person of color would be analyzed as being in the “target” group, whereas persons who self-identified as being white non-Hispanic would be analyzed as being in the “perpetrator” group. Each would: (a) self-report their own exposure to discrimination based on race/ethnicity as well as self-report their appraisal of their self-identified group’s exposure to discrimination, and (b) complete the B-IAT instruments for racial discrimination.

Given that the primary interest of this research was in implicit cognition, the order of these measures for each type of discrimination was fixed. That is, for each type of discrimination, participants first completed the two B-IATs and then the two explicit measures. However, to avoid order effects, the order within implicit and explicit measures was randomized across participants. Altogether, each experiment required about 10 min to complete, with only 2 of these 10 min needed for each B-IAT.

### Measures

#### Demographics

Participants completed a request for key demographic items relevant to each type of discrimination: sex, gender identity, sexual orientation, race/ethnicity, weight, and age. For these questions, respondents self-identified their characteristics at the time of the study (see response options in Table S1). For the age discrimination experiment, we additionally categorized participants as either a “young” (24–44) vs “old” (45–64) working-age adult, given evidence that workplace discrimination based on age, legally defined in the U.S. in relation to age 40 and older, is especially prevalent for U.S. workers age 45 and older [1, 39]. Participants completed also a request for nationality and education.

#### Brief implicit association test (B-IAT)

Implicit discrimination and attitudes were measured by means of a Target/Perpetrator Brief Implicit Association Test (Target/Perpetrator B-IAT) and a Good/Bad Brief Implicit Association Test (Good/Bad B-IAT), respectively.

In both B-IATs, participants were presented with words belonging to two attributes and two categories. The Target/Perpetrator B-IAT included words from the two attributes *Target of Discrimination* (e.g., victim and oppressed) and *Perpetrator of Discrimination* (e.g., perpetrator and abuser), while the Good/Bad B-IAT included words from the two attributes *Good* (e.g., love and pleasant) and *Bad* (e.g., hate and unpleasant). Categories for B-IATs differed on the basis of the social construct examined in each experiment. For example, in the racism experiment Target/Perpetrator B-IAT and Good/Bad B-IAT included words from the two categories *White People* (e.g., White and Caucasian) and *People of Color* (e.g., Black and Latinx), while in the sexism experiment Target/Perpetrator B-IAT and Good/Bad B-IAT included words from the two categories *Female* (e.g., Women and She) and *Male* (e.g., Men and He). The order of the two B-IATs was randomized between subjects. Stimuli used in the BIATs by experiment are presented in Table S2.

Both B-IATs followed the standard task procedure described by Sriram & Greenwald (2009). Participants were instructed to focus on just one category and one attribute. Stimuli appeared one at a time in the middle of the screen and participants categorized each stimulus as either belonging to one of the focal category or attribute (press the ‘i’ key) or not (press the ‘e’ key). If the participant made an error, a red “X” appeared below the stimulus and the trial continued until the correct key was pressed.

Each B-IAT included four blocks of 20 trials each. In each block, the first four trials were selected from the categories of interest (e.g., *White People* and *People of Color* in the racism experiment). The remaining 16 trials for each block alternated between categories of interest and attributes (i.e., *Target of Discrimination* and *Perpetrator of Discrimination* for the Target/Perpetrator B-IAT; *Good* and *Bad* for the Good/Bad B-IAT). All the blocks had the same focal attribute (i.e., *Target of Discrimination* for the Target/Perpetrator B-IAT and *Good* for the Good/Bad B-IAT) and alternated the focal category (e.g., *White People* and *People of Color* in the racism experiment) such that the same combination between attribute and focal category (e.g., *White People + Target of Discrimination* or *White People + Good*) appeared in blocks 1 and 3 and the other combination (e.g., *People of Color + Target of Discrimination* or *People of Color + Good*) in blocks 2 and 4. The order of the combinations between attribute and focal category was counterbalanced across subjects.

Target/Perpetrator B-IAT and Good/Bad B-IAT were preceded by two 20-trial warm-up blocks. In one block, participants were presented with flowers (e.g., orchid and lilac) and good words as the focal categories and insects (e.g., flea and centipede) and bad words as non-focal categories. In the other block, participants were presented with insects and good words as the focal categories and flowers and bad words as non-focal categories. The order of the two practice blocks was counterbalanced across subjects.

Scores for the Target/Perpetrator B-IAT and Good/Bad B-IAT were computed according to the recommended B-IAT algorithm described by Nosek et al. (2014). That is, we divided the difference in mean between the two B-IAT attribute-focal category conditions by the standard deviation of the latencies inclusive of the two conditions. Responses in the first four trials of each block and those slower than 10,000 ms were removed. Responses faster than 400 ms or slower than 2000 ms were recoded to 400 ms and to 2000 ms, respectively. Participants faster than 400 ms on more than 10% of trials were excluded as indicative of careless participation. 8.3% of the B-IAT sessions were removed based on these exclusion criteria.

In all experiments, positive scores in the Target/Perpetrator B-IAT and in the Good/Bad B-IAT indicated an implicit recognition of discrimination towards the target group and an implicit preference for the dominant group, respectively. For example, in the racism experiment, positive scores in the Target/Perpetrator B-IAT indicated an implicit recognition of discrimination towards People of Color, and positive scores in the Good/Bad B-IAT indicated an implicit preference for White People. Scores could range from + 2 to − 2, with zero indicating neutrality or no difference in association between attributes and social categories.

#### Self-reported items

Explicit discrimination and attitudes were measured using self-reported items. In particular, explicit discrimination was assessed by means of two questions evaluating explicit group discrimination (e.g., in the racism experiment, “How often do you feel that people of Color are discriminated against because of their race?”) and explicit individual discrimination (in the racism experiment, “How often do you feel that you, personally, have been discriminated against because of your race?”). Responses were obtained on a 4-point scale and coded as scores from 0 to 3 (i.e., 0 “Never”, 1 “Rarely”, 2 “Sometimes”, and 3 “Often”). Explicit attitudes were measured by means of a single item. Participants were asked to select which statement best described them from seven options. For example, for the racism experiment, the options were: (1) I strongly, (2) I moderately, (3) I slightly “prefer People of Color (e.g., Black, Latinx, and Asian) to White People”, (4) “I prefer People of Color (e.g., Black, Latinx, and Asian) and White People equally”, and (5) I slightly, (6) I moderately, and (7) I strongly “prefer White People to People of Color (e.g., Black, Latinx, and Asian)”. Responses were coded as scores from − 3 to + 3 with more positive scores indicating stronger preferences for a social group over the other one (e.g., in the racism experiment, more positive scores indicating stronger preferences for White People over People of Color).

All the explicit items by experiment are available in Table S3.

## Results

### Sample characteristics

The average age of all participants was 37.8 (*SD* = 9.9); 68.6% were White non-Hispanic people and 31.4% were people of Color; 69% were females and 31% were males; 76.1% were heterosexual people and 23.9% were lesbian, gay, bisexual, and queer (LGBQ) people; 90.7% were gender conforming people (63.7% women, 27.1% men) and 9.3% were gender minority people (0.9% trans women, 1.4% trans men, 7% genderfluid/non-binary); 2.6% were underweight, 52.8% were medium weight, 36.5% were overweight, and 8.1% were obese; 0.8% had some or less than high school education, 1.7% had a high school degree, 21.8% had some college education, 34.4% had a Bachelor of Arts or a Bachelor of Science degree, and 41.5% had an advanced degree. Sample demographics by experiment and participant group are reported in Table S1.

### Implicit and explicit discrimination

Implicit and explicit scores for discrimination, and correlations are displayed in Table 1. In the following, we discuss only the main results. A detailed description of all the results is reported in the Supplementary Material.

**Table 1:** Implicit and explicit scores for discrimination by experiment and participant group

														Implicit and Explicit Correlations	
Experiment	Participant Group	Target/Perpetrator B-IAT (Implicit Discrimination)				Explicit Group Discrimination				Explicit Individual Discrimination				Target/Perpetrator B-IAT and Explicit Group Discrimination	Target/Perpetrator B-IAT and Explicit Individual Discrimination
		N	M	SD	Group Comparison	N	M	SD	Group Comparison	N	M	SD	Group Comparison	r	r
**Racism**	**People of Color**	67	0.29**	0.42	**	77	2.51**	0.74	n.s.	77	1.87**	0.85	**	0.14	0.09
	**White**	74	0.00	0.36		78	2.69**	0.54		78	0.76**	0.72		0.26*	−0.24*
**Sexism**	**Female**	77	0.29**	0.41	n.s.	75	2.48**	0.69	n.s.	75	1.83**	0.76	**	0.16	0.28*
	**Male**	73	0.28**	0.31		75	2.28**	0.75		75	1.07**	0.81		0.20	0.17
**Heterosexism**	**LGBQ**	72	0.48**	0.46	**	75	2.79**	0.41	**	75	1.53**	0.79	**	−0.02	−0.10
	**Straight**	71	0.23**	0.41		78	2.53**	0.66		78	0.31**	0.61		−0.12	− 0.04
**Transphobia**	**Trans**	62	0.57**	0.39	**	76	2.88**	0.43	n.s.	76	1.88**	0.92	n.s.	−0.01	−0.04
	**Cis**	62	0.22**	0.42		80	2.74**	0.50		80	2.14**	0.90		0.09	0.00
**Ageism**	**Older**	42	0.28**	0.37	*	44	2.02**	0.70	n.s.	44	1.16**	0.81	n.s.	−0.13	−0.24
	**Younger**	79	0.13**	0.39		81	1.90**	0.78		81	1.33**	0.74		0.14	0.01
**Fatphobia**	**Heavy**	75	−0.01	0.43	n.s.	78	2.51**	0.60	n.s.	78	1.51**	0.89	**	0.14	−0.05
	**Not heavy**	72	−0.13**	0.37		78	2.47**	0.73		78	0.60**	0.87		0.02	0.00

B-IAT scores could range from + 2 to −2, whereas explicit scores could range from 0 to + 3. Positive scores in the B-IAT and explicit group discrimination indicated the presence of a recognition of discrimination towards the target group. Positive scores in explicit individual discrimination indicated the presence of a recognition of discrimination towards self as a member of a specific social group. Zero indicated no relative preference. Note. Group comparison refers to comparison of scores (mean values) across the participant groups for each experiment; *M* Mean, *SD* standard deviation; *p < 0.05 (2-sided); ** *p* < 0.01 (2-sided), *n.s* not significant

#### Implicit discrimination

Overall, results showed an implicit recognition of exposure to discrimination towards the target group in all the experiments and participant groups involving sex discrimination targeted at women, discrimination against sexual minorities, discrimination against gender minorities, and discrimination at older participants (age 45 years or more). Stronger implicit recognition of exposure to discrimination was observed among participants belonging to the target groups, i.e., LGBQ, transgender, and older.

The only exceptions were observed in the racism and fatphobia experiments. In the racism experiment, only people of Color showed an implicit recognition of exposure to discrimination towards the target group (i.e., *People of Color* + *Target of Discrimination/White People* + *Perpetrator of Discrimination* associations; *M* = 0.29, *SD* = 0.42, Cohen’s *d* = 0.69, *t*(66) = 5.780, *p* < 0.001, 95% C.I. [0.19, 0.40]), while White people were neutral (i.e., no significant association between social categories *People of Color* and *White people* and attributes *Target of Discrimination* and *Perpetrator of Discrimination*; *M* = 0.00, *SD* = 0.36, Cohen’s *d* = 0.00, *t*(73) = 0.096, *p* = 0.924, 95% C.I. [− 0.08, 0.09]) . In the fatphobia experiment, heavy participants showed no significant association between social categories *Fat people* and *Thin people* and attributes *Target of Discrimination* and *Perpetrator of Discrimination* (*M* = -0.01, *SD* = 0.43, Cohen’s *d* = − 0.02, *t*(74) = − 0.123, *p* = 0.903, 95% C.I. [− 0.11, 0.09]), while a slight implicit recognition of exposure to discrimination towards the dominant group was instead observed for not heavy participants (i.e., *Thin people + Target of Discrimination/ Fat people + Perpetrator of Discrimination* associations; *M* = -0.13, *SD* = 0.37, Cohen’s *d* = − 0.35, *t*(71) = − 2.990, *p* < 0.01, 95% C.I. [− 0.21, − 0.04]).

#### Explicit group-discrimination

Similar to the implicit data, explicit measures showed a recognition of exposure to discrimination towards the target group in all the experiments and participant groups. Differences in the strength of explicit group discrimination, however, between the targeted and perpetrator participant groups were observed in the heterosexism experiment. That is, LGBQ participants showed stronger explicit group discrimination (i.e., were more likely to report exposure to discrimination towards this group) than straight participants, *F*(1, 152) = 8.545, *p* < 0.01, *ηp*^*2*^ = 0.05.

#### Explicit individual discrimination

In each experiment, all participant groups self-reported exposure to discrimination towards themselves, i.e., reported an explicit individual discrimination. Differences in the strength of explicit individual discrimination, however, between the targeted and perpetrator participant groups were observed in the racism, sexism, heterosexism, and fatphobia experiments. That is, participants belonging to target groups showed stronger explicit individual discrimination than participants belonging to dominant groups. Specifically, in the racism experiment participants of Color showed stronger explicit individual discrimination than White participants, *F*(1, 154) = 77.346, *p* < 0.001, *ηp*^*2*^ = 0.34; in the sexism experiment, females showed stronger explicit individual discrimination than males, *F*(1, 149) = 35.068, *p* < 0.001, *ηp*^*2*^ = 0.19; in the heterosexism experiment, LGBQ participants reported stronger explicit individual discrimination than straight participants, *F*(1, 152) = 115.206, *p* < 0.001, *ηp*^*2*^ = 0.43; and in the fatphobia experiment, heavy participants showed stronger explicit individual discrimination than not heavy participants, *F*(1, 155) = 41.412, *p* < 0.001, *ηp*^*2*^ = 0.21.

#### Correlations between implicit and explicit measures of discrimination

A significant correlation between implicit scores and explicit group discrimination was observed only for White participants in the racism experiment (*r* = 0.26, *p* < 0.05). That is, White participants who showed stronger implicit discrimination also reported greater explicit group discrimination. With regards to the correlations between implicit scores and explicit individual discrimination, a significant effect emerged for White participants in the racism experiment (*r* = − 0.24, *p* < 0.05) and for females in the sexism experiment (*r* = 0.28, *p* < 0.05). That is, White participants who showed stronger implicit discrimination reported weaker explicit individual discrimination; and females who showed stronger implicit discrimination also reported greater explicit individual discrimination.

### Implicit and explicit attitudes

Implicit and explicit scores for attitudes, and correlations are displayed in Table 2. In the following, we discuss only the main results. A detailed description of all the results is reported in the Supplementary Material.

**Table 2:** Implicit and explicit scores for attitudes by experiment and participant group

										Implicit and Explicit Correlations
Experiment	Participant Group	Good/Bad B-IAT (Implicit Attitude)				Explicit Attitude				Good/Bad B-IAT and Explicit Attitude
		N	M	SD	Group Comparison	N	M	SD	Group Comparison	r
**Racism**	**People of Color**	74	0.09	0.50	**	77	−0.77**	1.06	**	0.10
	**White**	78	0.30**	0.42		77	0.06	0.7		0.32**
**Sexism**	**Female**	79	−0.59**	0.36	**	74	−0.23*	0.84	n.s.	0.17
	**Male**	72	−0.15**	0.37		73	−0.15	1.20		0.06
**Heterosexism**	**LGBQ**	72	−0.35**	0.47	**	75	−0.55**	1.02	**	0.31**
	**Straight**	74	0.16**	0.50		75	0.52**	1.07		0.40**
**Transphobia**	**Trans**	70	−0.36**	0.43	**	74	−1.35**	1.38	**	0.22
	**Cis**	77	0.14**	0.41		78	0.63**	1.16		0.31**
**Ageism**	**Older**	42	−0.12	0.40	**	40	−0.30*	0.91	**	−0.04
	**Younger**	80	0.10*	0.35		79	0.23	1.25		0.15
**Fatphobia**	**Heavy**	76	0.28**	0.44	*	77	0.42**	0.94	n.s.	0.13
	**Not heavy**	77	0.42**	0.45		76	0.68**	0.88		0.20

B-IAT scores could range from + 2 to −2, whereas explicit scores could range from −3 to + 3. Positive scores indicated a preference for the dominant group. Zero indicated no relative preference. Note. Group comparison refers to comparison of scores (mean values) across the participant groups for each experiment; *M* Mean, *SD* standard deviation; *p < 0.05 (2-sided); ** *p* < 0.01 (2-sided); *n.s* not significant

#### Implicit attitudes

Results for implicit attitudes varied by type of discrimination. In the heterosexism and transphobia experiments, results showed an in-group attitude for all participant groups. In contrast, in the racism, ageism, sexism, and fatphobia experiments not all the participant groups showed an in-group attitude. Specifically, in the racism experiment, an in-group attitude (i.e., *White People + Good/People of Color + Bad* associations) was observed only for White participants (*M* = 0.30, *SD* = 0.42, Cohen’s *d* = 0.71, *t*(77) = 6.404, *p* < 0.001, 95% C.I. [0.21, 0.40]), while participants of Color showed neutrality (*M* = 0.09, *SD* = 0.50, Cohen’s *d* = 0.18, *t*(73) = 1.617, *p* = 0.110, 95% C.I. [− 0.02, 0.21]). In the ageism experiment, an in-group attitude (i.e., *Adults older than 45 + Bad/Adults younger than 45 + Good* associations) was observed only for younger participants (*M* = 0.10, *SD* = 0.35, Cohen’s *d* = 0.29, *t*(79) = 2.552, *p* < 0.05, 95% C.I. [0.02, 0.18]), while older participants showed no significant associations (*M* = -0.12, *SD* = 0.40, Cohen’s *d* = 0.30, *t*(41) = − 1.907, *p* = 0.064, 95% C.I. [− 0.24, 0.01]. In the sexism experiment, both female (*M* = -0.59, *SD* = 0.36, Cohen’s *d* = − 1.64, *t*(78) = − 14.662, *p* < 0.001, 95% C.I. [− 0.67, − 0.51]) and male (*M* = -0.15, *SD* = 0.37, Cohen’s *d* = − 0.41, *t*(71) = − 3.363, *p* < 0.001, 95% C.I. [− 0.23, − 0.06]) participants showed *Female + Good/Male + Bad* associations, indicating a preference for females in both participant groups. In the fatphobia experiment both heavy (*M* = 0.28, *SD* = 0.44, Cohen’s *d* = 0.64, *t*(75) = 5.536, *p* < 0.001, 95% C.I. [0.18, 0.38]) and not heavy (*M* = 0.42, *SD* = 0.45, Cohen’s *d* = 0.93, *t*(76) = 8.158, *p* < 0.001, 95% C.I. [0.32, 0.52]) participants showed *Thin People + Good/Fat People + Bad* associations, indicating a preference for thin people in both participant groups.

Differences in the strength of implicit attitudes between participant groups were observed in the sexism and fatphobia experiments. That is, females showed stronger *Female + Good/Male + Bad* associations than males, *F*(1, 150) = 56.712, *p* < 0.001, *ηp*^*2*^ = 0.276, and heavy participants showed stronger *Thin People + Good/Fat People + Bad* associations than not heavy participants, *F*(1, 152) = 3.896, *p* < 0.05, *ηp*^*2*^ = 0.025.

#### Explicit attitudes

Results for explicit attitudes likewise varied by type of discrimination. Similar to the implicit results, in the heterosexism and transphobia experiments, all participant groups showed an in-group attitude. In contrast, an in-group attitude for all participant groups was not equally observed in the racism, ageism, sexism, and fatphobia experiments. In the racism experiment, an in-group preference was observed only for People of Color (*M* = -0.77, *SD* = 1.06, Cohen’s *d* = − 0.73, *t*(76) = − 6.328, *p* < 0.01, 95% C.I. [− 1.01, − 0.53], while White people reported to like People of Color and White people equally (*M* = 0.06, *SD* = 0.70, Cohen’s *d* = 0.09, *t*(76) = 0.820, *p* = 0.415, 95% C.I. [− 0.09, 0.22]). In the ageism experiment, an in-group attitude was observed only for older participants (*M* = -0.30, *SD* = 0.91, Cohen’s *d* = − 0.33, *t*(39) = − 2.082, *p* < 0.05, 95% C.I. [− 0.59, 0.01]), while younger participants showed no significant preferences (*M* = 0.23, *SD* = 1.25, Cohen’s *d* = 0.18, *t*(78) = 1.620, *p* = 0.109, 95% C.I. [− 0.05, 0.51]). In the sexism experiment females reported an in-group preference (*M* = -0.23, *SD* = 0.84, Cohen’s *d* = − 0.27, *t*(73) = − 2.362, *p* < 0.05, 95% C.I. [− 0.42, − 0.04], while males reported no significant preferences (*M* = -0.15, *SD* = 1.20, Cohen’s *d* = − 0.13, *t*(72) = − 1.075, *p* = 0.286, 95% C.I. [− 0.43, 0.13]). In the fatphobia experiment, both heavy (*M* = 0.42, *SD* = 0.94, Cohen’s *d* = 0.45, *t*(76) = 3.893, *p* < 0.001, 95% C.I. [0.20, 0.63]) and not heavy (*M* = 0.68, *SD* = 0.88, Cohen’s *d* = 0.77, *t*(75) = 6.758, *p* < 0.001, 95% C.I. [0.48, 0.89]) participants showed a preference for thin people.

#### Correlations between implicit and explicit attitudes

Significant correlations between implicit and explicit attitudes emerged for White participants in the racism experiment (*r* = 0.32, *p* < 0.01), for both LGBQ (*r* = 0.31, *p* < 0.01) and straight participants (*r* = 0.40, *p* < 0.001) in the heterosexism experiment, and for cisgender participants in the transphobia experiment (*r* = 0.31, *p* < 0.01). All these correlations were positive, indicating that stronger implicit attitudes in these participants were also associated with stronger explicit attitudes.

### Correlations between measures of discrimination and attitudes

In the following, we report only the significant correlations. All the correlations by experiment and participants group are reported in Table 3.

**Table 3:** Correlations between measures of discrimination and attitudes by experiment and participant group

			Measures of Discrimination		
Measures of Attitudes	Experiment	Participant Group	Target/Perpetrator B-IAT (Implicit Discrimination)	Explicit Group Discrimination	Explicit Individual Discrimination
			r	r	r
**Good/Bad B-IAT (Implicit Attitude)**	**Racism**	**People of Color**	−0.14	−0.15	−0.06
		**White**	−0.28*	−0.03	0.15
	**Sexism**	**Female**	−0.23*	−0.18	− 0.01
		**Male**	−0.20	−0.20	− 0.00
	**Heterosexism**	**LGBQ**	−0.27*	−0.23	− 0.14
		**Straight**	−0.18	−0.20	− 0.07
	**Transphobia**	**Trans**	−0.17	0.04	−0.14
		**Cis**	−0.12	0.16	−0.04
	**Ageism**	**Older**	−0.38*	0.22	0.14
		**Younger**	−0.11	− 0.10	−0.09
	**Fatphobia**	**Heavy**	−0.12	−0.16	0.03
		**Not heavy**	0.08	−0.10	−0.01
**Explicit Attitude**	**Racism**	**People of Color**	−0.21	−0.10	− 0.38**
		**White**	−0.06	−0.02	0.01
	**Sexism**	**Female**	−0.35**	−0.21	− 0.18
		**Male**	−0.04	−0.09	− 0.05
	**Heterosexism**	**LGBQ**	−0.10	−0.06	− 0.30**
		**Straight**	−0.23	−0.20	− 0.16
	**Transphobia**	**Trans**	0.09	−0.25*	−0.43**
		**Cis**	−0.29*	−0.21	− 0.28*
	**Ageism**	**Older**	0.04	−0.29	0.09
		**Younger**	−0.07	0.17	0.10
	**Fatphobia**	**Heavy**	0.03	0.15	0.01
		**Not heavy**	−0.19	0.20	0.16

*p < 0.05 (2-sided); ** *p* < 0.01 (2-sided)

#### Correlations between implicit measures (i.e., target/perpetrator B-IAT and good/bad B-IAT correlations)

Significant correlations were observed for White participants in the racism experiment (*r* = − 0.28, *p* < 0.05), for females in the sexism experiment (*r* = − 0.23, *p* < 0.05), for LGBQ participants in the heterosexism experiment (*r* = − 0.27, *p* < 0.05), and older participants in the ageism experiment (*r* = − 0.38, *p* < 0.05). All these correlations were negative, indicating that for these participants stronger implicit recognition of exposure to discrimination towards the target groups was associated with weaker preferences for the dominant groups.

#### Correlations between explicit measures (i.e., explicit group discrimination and explicit attitudes correlations, explicit individual discrimination and explicit attitudes correlations)

A significant correlation between explicit group discrimination and explicit attitudes emerged only for transgender participants in the transphobia experiment (*r* = − 0.25, *p* < 0.05), indicating that transgender participants who showed stronger explicit self-reported exposure to discrimination towards gender minority people reported weaker explicit preferences for gender conforming people.

Significant correlations between explicit individual discrimination and explicit attitudes were observed for People of Color in the racism experiment (*r* = − 0.38, *p* < 0.001), LGBQ participants in the heterosexism experiment (*r* = − 0.30, *p* < 0.01), and both transgender (*r* = − 0.43, *p* < 0.001) and cisgender (*r* = − 0.28, *p* < 0.01) participants in the transphobia experiment. All these correlations were negative, indicating that for these participants, stronger explicit recognition of exposure to discrimination towards themselves was associated with weaker explicit preferences for the dominant groups.

#### Correlations between implicit discrimination and explicit attitudes (i.e., target/perpetrator B-IAT and explicit attitudes correlations)

Significant correlations between implicit discrimination and explicit attitudes were observed for females in the sexism experiment, and cisgender participants in the transphobia experiment. These correlations were negative, indicating that for these participants stronger implicit recognition of exposure to discrimination for the target groups was associated with weaker explicit preferences for the dominant groups.

## Discussion

The present study is the first to employ the B-IAT to assess exposure to multiple types of discrimination. Specifically, we used the B-IAT to measure six types of discrimination based on race/ethnicity, sex, gender identity, sexual orientation, age, and weight.

Overall, we found implicit and explicit recognition of exposure to discrimination towards target groups. This result was observed both among individuals belonging to target groups and to dominant groups, but was stronger in the former. Implicit and explicit measures of discrimination tended to show no correlations. Similarly, no correlations were generally observed between implicit recognition of discrimination and implicit or explicit attitudes towards social groups of interest, indicating that implicit recognition of exposure to discrimination was not influenced by social preferences.

However, some exceptions emerged in experiments investigating discrimination in relation to race/ethnicity and weight. In the racism experiment, we found that White people, unlike people of Color, showed no implicit recognition of discrimination towards people of Color (i.e., Black, Asian, and Latinx). That is, no significant association of the categories *People of Color* and *White People* with the attributes *Target* and *Perpetrator* was observed. In contrast, a recognition of discrimination against people of Color emerged at the explicit level. In other words, results showed that although White people reported feeling that people of Color are discriminated against because of their race/ethnicity at a conscious level, no recognition of such discrimination was observed at an unconscious level. However, a low positive correlation was observed between implicit and explicit measures of discrimination, indicating that these measures assessed distinct but related constructs [40]. In addition, we found that for White people, the implicit measure of discrimination was also negatively associated with implicit attitudes, i.e. stronger pro-White attitudes reduced the likelihood of implicit recognition of discrimination towards people of Color. These results expand existing research on implicit recognition of discrimination based on race/ethnicity. They suggest that explicit statements by White people about exposure to discrimination among people of Color may not be matched to their implicit recognition of such discrimination.

Of note, in contrast to our findings, the three prior studies using implicit measures to assess discrimination towards Black people, whose participants were recruited over a decade ago (i.e., between 2007 and 2010), showed that both Black and White individuals show implicit associations indicating a recognition of discrimination towards Black people [23–26]. That is, discrimination towards Black people was recognized by both groups at an unconscious level. Here, we found instead that when implicit measures are used to assess the implicit recognition of discrimination towards people of Color (i.e., Black, Latinx, Asian), and not only towards Black people, White people show no implicit association. One possible reason for the difference in results observed in our study and the prior studies is that White people may show implicit recognition towards some specific race/ethnicity groups (e.g., Black people) but not towards other race/ethnicity groups that still belong to the category people of Color (e.g., Latinx and Asian). In the U.S., extensive research has documented both the specificities of anti-Black racism, as tied to enduring impacts of histories of enslavement, along with racism directed against other groups (e.g., American Indians/Native Americans in relation to histories of settler-colonialism, and racism against Latinx and Asian groups tied to histories of immigration) [41–43]. Future studies, in which implicit recognition of exposure to discrimination towards each specific race/ethnic groups (i.e., Black, Latinx and Asian) is measured, would be thus useful to quantify and compare discrimination against different social groups categorized in the U.S. as people of Color. An alternative hypothesis is that explicit and implicit measures of discrimination and also the relationships between these measures may be influenced by societal context, which is tied to changes in race relations and race politics, and it has been shown to influence measures of racial attitudes [44, 45]. Additionally, new research suggests that implicit biases may reflect structural inequalities, not just individual dispositions, and thus change as norms and practices of structural racism change [46]. It is indeed relevant to note that the first three studies that used implicit measures to assess exposure to racial discrimination were conducted during the early years of the Obama Administration, which supported a public discourse regarding racial equality (even as policies did not always follow suit). In contrast, the current study was conducted during the Trump Administration, whose policies and pronouncements have been associated with an increase in racial intolerance and hate crimes [47–51], along with partisan differences in expressing white grievance and racial resentment political views [52]. The impact of changing political discourse and policies about race relations and racial justice on measures of both explicit and implicit discrimination warrant further study.

Similarly, in the fatphobia experiment, we found different results on implicit and explicit measures of discrimination and among participant groups. Specifically, on explicit measures, both heavy and not heavy groups reported feeling that fat people are discriminated against because of their weight, while results for implicit measures differed between participant groups. That is, heavy individuals showed no implicit association between the categories *Thin People* and *Fat People* and the attributes *Target* and *Perpetrator*, while not heavy individuals showed slight *Thin People + Target/Fat People + Perpetrator* associations. In other words, although discrimination towards fat people was recognized at a conscious level, no recognition of this discrimination (heavy individuals) or opposite results (not heavy individuals) were observed at an unconscious level. These results are in line with previous studies using implicit and explicit measures to assess other psychological constructs based on weight, such as weight attitudes. These studies showed that implicit and explicit weight attitudes can display a large degree of dissociation or even opposite effects [53–55]. For example, it has been shown that preferences between overweight/obese and underweight people differed when assessed by means of explicit or implicit measures. That is, pro-underweight preferences were observed at the explicit level, while pro-overweight/obese preferences were found at the implicit level [54]. The lack of an implicit recognition of discrimination towards fat people found in the present study may reflect the assumption that overweight/obese people are responsible for their condition and that thus they cannot be considered victims of discrimination. Studies have shown indeed that obese/overweight people are perceived as lacking self-discipline and control and, for this reason, blamed for their weight [56–58]. This belief may thus lead individuals to view fat people as not victims but even perpetrators of their condition and potential discrimination. This effect may emerge only on implicit measures as they are less influenced by intentional and social desirability processes. Indeed, although in Western societies, it is partially acceptable to denigrate obese and overweight people [59], individuals may avoid reporting their evaluations at an explicit level because they may be viewed negatively from others.

Taken together, our findings have important applications for health research, in particular for those investigating the effects of discrimination on health. Our results show that for specific types of discrimination, implicit and explicit measures can display diverging results, indicating that these instruments can provide different information (but both valuable) about recognition of discrimination. Previous studies showed that implicit constructs (e.g., attitudes and stereotypes) are pervasive [55, 60, 61] and predict variations in behavior across a variety of topics, in many cases above and beyond those assessed by means of explicit measures [62–64] (for a meta-analytical comparison of the predictive power of implicit and explicit measures see Kurdi et al. [65]). For example, it has been shown that implicit (but not explicit) race/ethnicity attitudes predicted physicians’ decisions to provide more thrombolysis recommendations for White than Black patients with acute coronary syndromes [66]. Implicit measures of discrimination may thus predict health outcomes more accurately than explicit measures, which may underestimate the toll of discrimination on health. Further studies that include assessment of health outcomes (e.g., psychological distress, sleep disorders, and harmful coping behaviors) will be thus important to determine how the implicit recognition of discrimination found here for multiple types of social groups might contribute to health inequities.

In addition, our results have relevant implications for research in social cognition and for scientists interested in investigating discrimination as a psychological, cultural, and social phenomenon. For example, further research using the B-IAT may evaluate whether the implicit recognition of discrimination differs by region of the U.S. or world, and is influenced by brief interventions or specific contextual factors [53, 67–71]. Recent research indeed showed that psychological constructs measured by means of implicit measures, such as social attitudes or stereotypes, can differ by geographic region and be associated with the societal context, including the complexities of political, cultural, and economic context [53, 72]. Together, these societal features can powerfully shape the beliefs and behavior of its members [73]. This novel study provides a brief and time-efficient tool to measure multiple types of implicit discrimination, including those that have not been conventionally measured (e.g., transphobia). The B-IAT improves measurement of discrimination at the implicit level and offers a sound methodology for subsequent studies. Future research would benefit from incorporating both implicit and explicit measures of discrimination.

Although the present study provides new relevant insights concerning multiple types of discrimination, some limitations should be noted. The samples used in our study may not represent a random selection of the general population. Selection biases might be present because the sample was comprised of people who learned about the Project Implicit website, volunteered to participate, had some interest in studies on implicit social cognition. Yet, we cannot identify any plausible reason why variation in selection biases across participants would explain the results found in our experiments. In addition, it is important to point out that by recruiting participants online through the Project Implicit website, samples are far more varied in ethnicity/race, age, class, and education level than most studies conducted in a specific location such as employees of a single hospital or students from a single university who also occupy a narrow age range. Nonetheless, replication with other sampling contexts will be useful to increase confidence in the observed results.

## Conclusions

The present study provides evidence that the B-IAT is a valuable tool to assess implicit recognition of discrimination. In particular, we showed that the B-IAT can quickly measure multiple types of discrimination - such as discrimination based on race/ethnicity, sex, gender identity, age, sexual orientation, and weight – reducing the time required for administration of the standard IAT. These results have important implications for the use of implicit measures to assess exposure to discrimination, including in large-scale population-based studies (where time is often of the essence for any given set of questions). Our results show that the B-IAT has several advantages: it is a flexible tool and thus can be potentially used to evaluate any kind of discrimination; it can be administrated quickly (around 2 min versus the 15 min required for the IAT), enabling a time-efficient assessment of discrimination; and it can also be administrated remotely (i.e., via the web), favoring data collection and access to different populations. In addition, it is important to note that the B-IAT as the standard IAT requires low-tech equipment as it can be readily administrated on mobile devices, tablets, or computers.

## Supplementary Information

**Additional file 1.**

## Data Availability

All the data and materials are available from the corresponding author on reasonable request.

## Abbreviations

B-IAT: Brief-Implicit Association Test
IAT: Implicit Association Test
LGBQ: Lesbian, Gay, Bisexual, and Queer
PGDD: Person-Group Discrimination Discrepancy
U.S.: United States

## Footnotes

1 In the present manuscript, we use the word “sex” given U.S. legal recognition of “sex discrimination” and the construct of “sexism,” both of which typically presume “sex” is a binary biological variable; that said we recognize that “sex discrimination” is inherently linked to, if not identical with, gender-based discrimination, and we further recognize that neither biological sex nor socially-defined “gender” are simply binaries [1, 17, 34, 35].
