# Supplementary material for: The target/perpetrator brief-implicit association test (B-IAT): an implicit instrument for efficiently measuring discrimination based on race/ethnicity, sex, gender identity, sexual orientation, weight, and age
Source: BMC Public Health. 2021 Jan 19;21:158. doi: 10.1186/s12889-021-10171-7 (PMC7814653; doi:10.1186/s12889-021-10171-7)
Supplement: Supplementary file 1 — Additional file 1. [file 12889_2021_10171_MOESM1_ESM.docx]

**Supporting Information**

The target/perpetrator Brief-Implicit Association Test (B-IAT): An implicit instrument for efficiently measuring discrimination based on race/ethnicity, sex, gender identity, sexual orientation, weight, and age

Maddalena Marini^1^, Pamela D. Waterman^2^, Emry Breedlove^2^, Jarvis T. Chen^2^, Christian Testa^2^, Sari L. Reisner^2^, Dana J. Pardee^3^, Kenneth H. Mayer^3^, & Nancy Krieger^2^

^1^ Istituto Italiano di Tecnologia, Ferrara, FE, Italy

^2^ Harvard T.H. Chan School of Public Health, Boston, MA, USA

^3^ The Fenway Institute, Boston, MA, USA

|  | **Experiment and Participant Group** | | | | | | | | | | | |  |
| --- | --- | --- | --- | --- | --- | --- | --- | --- | --- | --- | --- | --- | --- |
| **Demographics** | **Racism** | | **Sexism** | | **Heterosexism** | | **Transphobia** | | **Ageism** | | **Fatphobia** | | **Total** |
|  | **People**  **of color** | **White Non- Hispanic** | **Women** | **Men** | **LGBQ** | **Straight** | **Transgender** | **Cisgender** | **Older**  **45-64 years** | **Younger**  **25-44 years** | **Heavy** | **Not Heavy** |  |
|  | **N = 112** | **N = 91** | **N = 84** | **N = 78** | **N = 77** | **N = 82** | **N = 79** | **N = 84** | **N = 47** | **N = 87** | **N = 80** | **N = 83** | **N=984** |
| **Sex: %** |  |  |  |  |  |  |  |  |  |  |  |  |  |
| Female | 76.6 | 80.0 | 100.0 | 0 | 75.3 | 75.6 | 58.8 | 64.3 | 72.3 | 73.3 | 72.5 | 79.5 | 69.0 |
| Male | 23.4 | 20.0 | 0 | 100.0 | 24.7 | 24.4 | 41.2 | 35.7 | 27.7 | 26.7 | 27.5 | 20.5 | 31.0 |
| **Gender Identity: %** |  |  |  |  |  |  |  |  |  |  |  |  |  |
| Woman | 75.9 | 78.0 | 98.8 | 2.6 | 74.0 | 75.6 | 0 | 63.1 | 72.3 | 73.6 | 70.9 | 79.5 | 63.7 |
| Man | 24.1 | 19.8 | 0 | 93.6 | 23.4 | 24.4 | 0 | 36.9 | 27.7 | 26.4 | 27.8 | 20.5 | 27.1 |
| Trans woman | 0 | 0 | 0 | 0 | 1.3 | 0 | 8.9 | 0 | 0 | 0 | 0 | 0 | 0.9 |
| Trans man | 0 | 0 | 0 | 1.3 | 0 | 0 | 15.2 | 0 | 0 | 0 | 0 | 0 | 1.4 |
| Genderfluid/non-binary | 0 | 2.2 | 1.2 | 2.6 | 1.3 | 0 | 75.9 | 0 | 0 | 0 | 1.3 | 0 | 7.0 |
| **Race/ethnicity: %*** |  |  |  |  |  |  |  |  |  |  |  |  |  |
| Person of color | 100.0 | 0 | 34.1 | 35.1 | 29.3 | 24.7 | 16.9 | 21.4 | 31.9 | 32.2 | 25.3 | 25.6 | 31.4 |
| White, non-Hispanic | 0 | 100.0 | 65.9 | 64.9 | 70.7 | 75.3 | 83.1 | 78.6 | 68.1 | 67.4 | 74.7 | 74.4 | 68.6 |
| **Weight: %** |  |  |  |  |  |  |  |  |  |  |  |  |  |
| Underweight | 0 | 5.5 | 4.8 | 2.6 | 3.9 | 2.4 | 3.8 | 3.6 | 0 | 3.5 | 0 | 1.2 | 2.6 |
| Medium weight | 61.3 | 44.0 | 45.8 | 53.8 | 57.1 | 57.3 | 56.4 | 46.4 | 53.2 | 59.3 | 0 | 98.8 | 52.8 |
| Overweight | 32.4 | 42.9 | 36.1 | 37.2 | 31.2 | 31.7 | 29.5 | 40.5 | 40.4 | 33.7 | 82.5 | 0 | 36.5 |
| Obese | 6.3 | 7.7 | 13.3 | 6.4 | 7.8 | 8.5 | 10.3 | 9.5 | 6.4 | 3.5 | 17.5 | 0 | 8.1 |
| **Sexual orientation: %** |  |  |  |  |  |  |  |  |  |  |  |  |  |
| LGBQ | 13.0 | 7.8 | 7.3 | 17.3 | 100.0 | 0 | 87.0 | 13.1 | 8.7 | 9.3 | 15.0 | 8.6 | 23.9 |
| Heterosexual or  straight | 87.0 | 92.2 | 92.7 | 82.7 | 0 | 100.0 | 13.0 | 86.9 | 91.3 | 90.7 | 85.0 | 91.4 | 76.1 |
| **Age:** |  |  |  |  |  |  |  |  |  |  |  |  |  |
| **Mean** | 36.03 | 39.52 | 35.48 | 38.13 | 35.14 | 36.62 | 32.48 | 39.02 | 50.62 | 33.14 | 38.19 | 39.37 | 37.8 |
| **SD** | 11.13 | 11.17 | 10.19 | 10.53 | 11.59 | 11.41 | 9.93 | 11.60 | 4.70 | 5.61 | 9.54 | 11.34 | 9.9 |
| **Education: %** |  |  |  |  |  |  |  |  |  |  |  |  |  |
| Some or less  than high school | 1.8 | 0 | 0 | 1.3 | 0 | 0 | 1.3 | 1.2 | 0 | 3.5 | 0 | 0 | 0.8 |
| High School | 2.8 | 1.1 | 0 | 2.6 | 2.6 | 1.2 | 1.3 | 1.2 | 0 | 1.2 | 1.3 | 4.8 | 1.7 |
| Some college | 22.9 | 22.0 | 26.8 | 23.4 | 14.3 | 28.0 | 22.8 | 22.6 | 17.0 | 19.8 | 25.0 | 16.9 | 21.8 |
| BA/BS | 35.8 | 34.1 | 25.6 | 33.8 | 41.6 | 40.2 | 30.4 | 35.7 | 29.8 | 44.2 | 27.5 | 33.7 | 34.4 |
| Advanced degree | 36.7 | 42.9 | 47.6 | 39.0 | 41.6 | 30.5 | 44.3 | 39.3 | 53.2 | 31.4 | 46.3 | 44.6 | 41.5 |

**Table S1.** **Summary of sample characteristics by experiment and participant group.**

Note. Sample size (N) and demographics refer to data of participants who completed at least one of the four measures used in each experiment (i.e., Target/Perpetrator B-IAT, Good/Bad B-IAT, self-reported items assessing explicit discrimination and self-reported item assessing explicit attitudes). Sex refers to sex assigned at birth. All participants were U.S. citizens and residents, aged between 25 to 64 years. Percentages refer to percent distribution with no missing data.

| **Demographics** | **Item** |
| --- | --- |
| Race/Ethnicity | Do you consider yourself to be a person of color or a White, non-Hispanic person?   - Person of Color - White, non-Hispanic |
| Sex | What is your sex at birth?   - Male - Female |
| Gender Identity | Do you consider yourself to be a woman, trans woman, man, trans man, or genderfluid/non-binary?   - Woman - Man - Trans woman - Trans man - Genderfluid/non-binary |
| Sexual Orientation | Do you consider yourself to be LGBQ or heterosexual/straight?   - LGBQ - Heterosexual or straight |
| Weight | Currently, are you underweight, medium weight, overweight or obese?   - Underweight - Medium weight - Overweight - Obese |
| Age | How old are you?   - [response options from 25 to 65] |

**Table S1. Demographic items used for assessing race/ethnicity, sex, gender identity, sexual orientation, weight and age.**

| **B-IAT** |  | | **B-IAT Attributes** | **Stimuli** |
| --- | --- | --- | --- | --- |
| Target/Perpetrator |  | | Target of Discrimination | Target, Victim, Oppressed |
|  |  | | Perpetrator of Discrimination | Perpetrator, Abuser, [3^rd^ term]  Note: the 3rd term varied by type of discrimination and was either: Racist; Sexist; Homophobic; Transphobic; Fatphobic; Ageist |
| Good/Bad |  | | Good | Love, Pleasant, Great, Wonderful |
|  |  | | Bad | Hate, Unpleasant, Awful, Terrible |
|  | | **Experiment** | **B-IAT Categories** | **Stimuli** |
|  | | Racism | White People | White, Euro-American, Caucasian |
|  |  |  | People of Color | Black, Asian, Latinx |
|  | Sexism | | Male | Men, He, His |
|  |  | | Female | Women, She, Her |
|  | Transphobia | | Gender Conforming | Female, Male, Cis-gender |
|  |  | | Gender Minority | Transgender, Gender-fluid, Non-binary |
|  | Heterosexism | | Heterosexual People | Straight, Heterosexual, Hetero |
|  |  | | Sexual minority People | Lesbian, Gay, Bisexual |
|  | Ageism | | Adults younger than 45 | Mid-20s, Mid-30s, Early-40s |
|  |  | | Adults older than 45 | Late-40s, Mid-50s, Mid-60s |
|  | Fatphobia | | Fat people | Overweight, Fat, Obese |
|  |  | | Thin People | Slim, Trim, Thin |

**Table S2.** **Stimuli used in the B-IATs by experiment.**

Note. Attributes in the Target/Perpetrator B-IAT and Good/Bad B-IAT were the same in all the experiments, while categories differed on the basis of the construct examined in each experiment. For example in the racism experiment, Target/Perpetrator B-IAT and Good/Bad B-IAT included words from the two categories *White People* (e.g., White and Caucasian) and *People of Color* (e.g., Black and Latinx), while in the sexism experiment Target/Perpetrator B-IAT and Good/Bad B-IAT included words from the two categories *Female* (e.g., Women and She) and *Male* (e.g., Men and He).

|  | **Item** | | |
| --- | --- | --- | --- |
| **Experiment** | **Explicit attitude** | **Explicit group discrimination** | **Explicit Individual discrimination** |
| **Racism** | Which statement best describes you?   - I strongly prefer People of Color (e.g., Black, Latinx, and Asian) to White people. - I moderately prefer People of Color (e.g., Black, Latinx, and Asian) to White people. - I slightly prefer People of Color (e.g., Black, Latinx, and Asian) to White people. - I like People of Color (e.g., Black, Latinx, and Asian) and White people equally. - I slightly prefer White people to People of Color (e.g., Black, Latinx, and Asian). - I moderately prefer White people to People of Color (e.g., Black, Latinx, and Asian). - I strongly prefer White people to People of Color (e.g., Black, Latinx, and Asian). | How often do you feel that racial/ethnic groups who are not White, such as Black, Latinx, and Asian, are discriminated against because of their race/ethnicity??   - Never - Rarely - Sometimes - Often | How often do you feel that you, personally, have been discriminated against because of your race, ethnicity, or color?   - Never - Rarely - Sometimes - Often |
| **Sexism** | Which statement best describes you?   - I strongly prefer people who are women to people who are men. - I moderately prefer people who are women to people who are men. - I slightly prefer people who are women to people who are men. - I like people who are women and people who are men equally. - I slightly prefer people who are men to people who are women. - I moderately people who are men to people who are women. - I strongly prefer people who are men to people who are women. | How often do you feel that women are discriminated against because of their gender?   - Never - Rarely - Sometimes - Often | How often do you feel that you, personally, have been discriminated against because of your gender?   - Never - Rarely - Sometimes - Often |
| **Heterosexism** | Which statement best describes you?   - I strongly prefer sexual minority people (e.g., lesbian, gay, bisexual) to heterosexual people. - I moderately prefer sexual minority people (e.g., lesbian, gay, bisexual) to heterosexual people. - I slightly prefer sexual minority people (e.g., lesbian, gay, bisexual) to heterosexual people. - I like sexual minority people (e.g., lesbian, gay, bisexual) and heterosexual people equally. - I slightly prefer heterosexual people to sexual minority people (e.g., lesbian, gay, bisexual). - I moderately prefer heterosexual people to sexual minority people (e.g., lesbian, gay, bisexual). - I strongly prefer heterosexual people to sexual minority people (e.g., lesbian, gay, bisexual). | How often do you feel that sexual minorities, such as lesbian, gay, and bisexual, are discriminated against because of their sexual orientation?   - Never - Rarely - Sometimes - Often | How often do you feel that you, personally, have been discriminated against because of your sexual orientation?   - Never - Rarely - Sometimes - Often |
| **Transphobia** | Which statement best describes you?   - I strongly prefer gender minority people (i.e., transgender, gender-fluid, non-binary) to gender conforming people (i.e., women, men, cis-gender). - I moderately prefer gender minority people (i.e., transgender, gender-fluid, non-binary) to gender conforming people (i.e., women, men, cis-gender). - I slightly prefer gender minority people (i.e., transgender, gender-fluid, non-binary) to gender conforming people (i.e., women, men, cis-gender). - I like gender minority people (i.e., transgender, gender-fluid, non-binary) and gender conforming people (i.e., women, men, cis-gender) equally. - I slightly prefer gender conforming people (i.e., women, men, cis-gender) to gender minority people (i.e., transgender, gender-fluid, non-binary). - I moderately prefer gender conforming people (i.e., women, men, cis-gender) to gender minority people (i.e., transgender, gender-fluid, non-binary). - I strongly prefer gender conforming people (i.e., women, men, cis-gender) to gender minority people (i.e., transgender, gender-fluid, non-binary). | How often do you feel that gender minority people (i.e., transgender, gender-fluid, non-binary) are discriminated against because of their gender identity?   - Never - Rarely - Sometimes - Often | How often do you feel that you, personally, have been discriminated against because of your gender identity?   - Never - Rarely - Sometimes - Often |
| **Ageism** | Which statement best describes you?   - I strongly prefer adults older than 45 (e.g., late-40s, mid-50s, mid-60s) to adults younger than 45 (e.g., mid-20s, mid-30s, early-40s). - I moderately prefer adults older than 45 (e.g., late-40s, mid-50s, mid-60s) to adults younger than 45 (e.g., mid-20s, mid-30s, early-40s). - I slightly prefer adults older than 45 (e.g., late-40s, mid-50s, mid-60s) to adults younger than 45 (e.g., mid-20s, mid-30s, early-40s). - I like adults older than 45 (e.g., late-40s, mid-50s, mid-60s) and adults younger than 45 (e.g., mid-20s, mid-30s, early-40s) equally. - I slightly prefer adults younger than 45 (e.g., mid-20s, mid-30s, early-40s) to adults older than 45 (e.g., late-40s, mid-50s, mid-60s). - I moderately prefer adults younger than 45 (e.g., mid-20s, mid-30s, early-40s) to adults older than 45 (e.g., late-40s, mid-50s, mid-60s). - I strongly prefer adults younger than 45 (e.g., mid-20s, mid-30s, early-40s) to adults older than 45 (e.g., late-40s, mid-50s, mid-60s). | How often do you feel that adults older than 45 (e.g., late-40s, mid-50s, mid-60s) are discriminated against because of their age?   - Never - Rarely - Sometimes - Often | How often do you feel that you, personally, have been discriminated against because of your age?   - Never - Rarely - Sometimes - Often |
| **Fatphobia** | Which statement best describes you?   - I strongly prefer Fat people to Thin people. - I moderately prefer Fat people to Thin people. - I slightly prefer Fat people to Thin people. - I like Fat people and Thin people equally. - I slightly prefer Thin people to Fat people. - I moderately prefer Thin people to Fat people. - I strongly prefer Thin people to Fat people. | How often do you feel that fat people are discriminated against because of their weight?   - Never - Rarely - Sometimes - Often | How often do you feel that you, personally, have been discriminated against because of a heavy weight?   - Never - Rarely - Sometimes   Often |

**Table S3. Explicit items by experiment.**

**Results**

**Implicit discrimination.** In the sexism experiment both female (*M*=0.29, *SD*=0.41, Cohen’s *d*=0.71, *t*(76)=6.291, *p*<0.001, 95% C.I. [0.20, 0.39]) and male (*M*=0.28, *SD*=0.31, Cohen’s *d*=0.90 , *t*(72)=7.606, *p*<0.001, 95% C.I. [0.20, 0.35]) participants showed *Female+Target of Discrimination/Male+Perpetrator of Discrimination* associations. In the heterosexism experiment, both LGBQ (*M*=0.48, *SD*=0.46, Cohen’s *d*=1.04, *t*(71)=9.152, *p*<0.001, 95% C.I. [0.38, 0.58]) and straight participants (*M*=0.23, *SD*=0.41, Cohen’s *d*=0.56, *t*(70)=4.660, *p*<0.001, 95% C.I. [0.13, 0.33]) showed *Sexual Minority People+Target of Discrimination/Heterosexual People+Perpetrator of Discrimination* associations. In the transphobia experiment, both transgender (*M*=0.57, *SD*=0.39, Cohen’s *d*=1.46, *t*(61)=11.684, *p*<0.001, 95% C.I. [0.47, 0.67]) and cisgender (*M*=0.22, *SD*=0.42, Cohen’s *d*=0.52, *t*(61)=4.238, *p*<0.001, 95% C.I. [0.12, 0.33]) participants showed *Gender Minority+Target of Discrimination/Gender Conforming+Perpetrator of Discrimination* associations. In the ageism experiment, both older (aged 45-65 years; *M*=0.28, *SD*=0.37, Cohen’s *d*=0.76, *t*(41)=4.994, *p*<0.001, 95% C.I. [0.17, 0.40]) and younger (aged 25-44 years; *M*=0.13, *SD*=0.39, Cohen’s *d*=0.33, *t*(78)=3.051, *p*<0.01, 95% C.I. [0.05, 0.22]) participants showed *Adults older than 45+Target of Discrimination/Adults younger than 45+Perpetrator of Discrimination* associations.

Stronger implicit recognition of exposure to discrimination was observed among participants belonging to the target groups. That is, LGBQ participants showed stronger *Sexual Minority People+Target of Discrimination/Heterosexual People+Perpetrator of Discrimination* associations than straight participants, *F*(1, 142)=12.312, *p*<0.001, *ηp²*=0.08; transgender participants showed stronger *Gender Minority+Target of Discrimination/Gender Conforming+Perpetrator of Discrimination* associations than cisgender participants, *F*(1, 123)=23.244, *p*<0.001, *ηp²*=0.16; older participants showed stronger *Adults older than 45+Target of Discrimination*/*Adults younger than 45+Perpetrator of Discrimination* associations than younger participants, *F*(1, 120)=4.239, *p*<0.05, *ηp²*=0.03.

**Explicit Group-discrimination.** In the racism experiment, both participants of Color (*M*=2.51, *SD*=0.74, Cohen’s *d*=3.39, *t*(76)=29.855, *p*<0.001, 95% C.I. [2.34, 2.67]) and White (*M*=2.69, *SD*=0.54, Cohen’s *d*=4.98 , *t*(77)=43.875, *p*<0.001, 95% C.I. [2.57, 2.81]) participants reported to feel that people of Color are discriminated against because of their race/ethnicity. In the sexism experiment, both male (*M*=2.28, *SD*=0.75, Cohen’s *d*=3.04, *t*(74)=26.488, *p*>0.001, 95% C.I. [2.11, 2.45]) and female (*M*=2.48, *SD*=0.69, Cohen’s *d*=3.59, *t*(74)=31.355, *p*>0.001, 95% C.I. [2.32,2.64]) participants reported to feel that females are discriminated against because of their sex. In the heterosexism experiment, both LGBQ (*M*=2.79, *SD*=0.41, Cohen’s *d*=6.80, *t*(74)=58.516, *p*>0.001, 95% C.I. [2.69, 2.88]) and straight (*M*=2.53, *SD*=0.66, Cohen’s *d*=3.83, *t*(77)=33.843, *p*>0.001, 95% C.I. [2.38, 2.67]) participants reported to feel that sexual minority people are discriminated against because of their sexual orientation. In the transphobia experiment, both transgender (*M*=2.88, *SD*=0.43, Cohen’s *d*=6.70, *t*(75)=58.281, *p*>0.001, 95% C.I. [2.78, 2.98]) and cisgender (*M*=2.74, *SD*=0.50, Cohen’s *d*=5.48, *t*(79)=49.299, *p*>0.001, 95% C.I. [2.63, 2.85]) participants reported to feel that gender minority people are discriminated against because of their gender identity. In the ageism experiment, both older (*M*=2.02, *SD*=0.70, Cohen’s *d*=2.89, *t*(43)=19.210, *p*>0.001, 95% C.I. [1.81, 2.24]) and younger (*M*=1.90, *SD*=0.78, Cohen’s *d*=2.44, *t*(80)=21.817, *p*>0.001, 95% C.I. [1.73, 2.07]) participants reported to feel that adults older than 45 are discriminated against because of their age. In the fatphobia experiment, both heavy (*M*=2.51, *SD*=0.60, Cohen’s *d*=4.18, *t*(77)=37.144, *p*>0.001, 95% C.I. [2.38, 2.65]) and not heavy (*M*=2.47, *SD*=0.73, Cohen’s *d*=3.38, *t*(77)=29.785, *p*>0.001, 95% C.I. [2.31, 2.64]) participants reported to feel that fat people are discriminated against because of their weight.

**Explicit Individual discrimination.** In the racism experiment, both participants of Color (*M*=1.87, *SD*=0.85, Cohen’s *d*=2.20, *t*(76)=19.343, *p*<0.001, 95% C.I. [1.68, 2.06]) and White (*M*=0.76, *SD*=0.72, Cohen’s *d*=1.06, *t*(77)=9.226, *p*<0.001, 95% C.I. [0.59, 0.91]) participants reported to feel personally discriminated against because of their race/ethnicity. In the sexism experiment, both male (*M*=1.07, *SD*=0.81, Cohen’s *d*=1.32, *t*(74)=11.391, *p*>0.001, 95% C.I. [0.88, 1.25]) and female (*M*=1.83, *SD*=0.76, Cohen’s *d*=2.41, *t*(74)=20.814, *p*>0.001, 95% C.I. [1.65, 2.00]) participants reported to feel personally discriminated against because of their sex. In the heterosexism experiment, both LGBQ (*M*=1.53, *SD*=0.79, Cohen’s *d*=1.94, *t*(74)=16.722, *p*>0.001, 95% C.I. [1.35, 1.72]) and straight (*M*=0.31, *SD*=0.61, Cohen’s *d*=0.51, *t*(77)=4.458, *p*>0.001, 95% C.I. [0.17, 0.45]) participants reported to feel personally discriminated against because of their sexual orientation. In the transphobia experiment, both transgender (*M*=1.88, *SD*=0.92, Cohen’s *d*=2.04, *t*(75)=17.766, *p*>0.001, 95% C.I. [1.67, 2.09]) and cisgender (*M*=2.14, *SD*=0.90, Cohen’s *d*=2.38, *t*(79)=21.327, *p*>0.001, 95% C.I. [1.94, 2.34]) participants reported to feel personally discriminated against because of their gender identity. In the ageism experiment, both older (*M*=1.16, *SD*=0.81, Cohen’s *d*=1.43, *t*(43)=9.547, *p*>0.001, 95% C.I. [0.91, 1.40]) and younger (*M*=1.33, *SD*=0.74, Cohen’s *d*=1.80, *t*(80)=16.181, *p*>0.001, 95% C.I. [1.17, 1.50]) participants reported to feel personally discriminated against because of their age. In the fatphobia experiment, both heavy (*M*=1.51, *SD*=0.89, Cohen’s *d*=1.70, *t*(77)=14.952, *p*>0.001, 95% C.I. [1.31, 1.71]) and not heavy (*M*=0.60, *SD*=0.87, Cohen’s *d*=0.70, *t*(77)=6.096, *p*>0.001, 95% C.I. [0.41, 0.80]) participants reported to feel personally discriminated against because of their weight.

**Implicit attitudes.** In the heterosexism experiment, LGBQ participants showed *Sexual Minority People+Good/Heterosexual People+Bad* associations (*M*=-0.35, *SD*=0.47, Cohen’s *d*=-0.74, *t*(71)=-6.314, *p*<0.001, 95% C.I. [-0.46, -0.24]), and straight participants showed *Sexual Minority People+Bad/Heterosexual People+Good* associations (*M*=0.16, *SD*=0.50, Cohen’s *d*=0.32, *t*(73)=2.731, *p*<0.01, 95% C.I. [0.04, 0.27]); and in the transphobia experiment, transgender participants showed *Gender Minority+Good/Gender Conforming+Bad* associations (*M*=-0.36, *SD*=0.43, Cohen’s *d*=-0.84, *t*(69)=-6.944, *p*<0.001, 95% C.I. [-0.46, -0.25) and cisgender participants showed *Gender Minority+Bad/Gender Conforming+Good* associations (*M*=0.14, *SD*=0.41, Cohen’s *d*=0.34, *t*(76)=2.967, *p*<0.01, 95% C.I. [0.05, 0.23]).

**Explicit attitudes.** In the heterosexism experiment, LGBQ participants reported preferences for sexual minority people (*M*=-0.55, *SD*=1.02, Cohen’s *d*=-0.54, *t*(74)=-4.654, *p*<0.001, 95% C.I. [-0.78, -0.31]), while straight participants showed preferences for heterosexual people (*M*=0.52, *SD*=1.07, Cohen’s *d*=0.49, *t*(74)=4.209, *p*<0.001, 95% C.I. [0.27, 0.77]); in the transphobia experiment, transgender participants preferred gender minority people (*M*=-1.35, *SD*=1.38, Cohen’s *d*=-0.98, *t*(73)=-8.428, *p*<0.001, 95% C.I. [-1.67, -1.03]), while cisgender participants showed preferences for gender conforming people (*M*=0.63, *SD*=1.16, Cohen’s *d*=0.54, *t*(77)=4.769, *p*<0.001, 95% C.I. [0.37, 0.89]).
